# CD44–Hyaluronan-Dependent Monocyte Rolling

**DOI:** 10.3390/ijms27125358

**Published:** 2026-06-13

**Authors:** Marcus Hubbe, Robert H. Eibl

**Affiliations:** 1 Laboratory of Immunology and Vascular Biology, Department of Pathology, Stanford University, Stanford, CA 94305, USA; 2 Center for Molecular Biology and Medicine, Veterans Affairs Health Care System, Palo Alto, CA 94304, USA; 3 I. L. Weissman Laboratory, Department of Pathology, Stanford University, Stanford, CA 94305, USA

**Keywords:** CD44, hyaluronan (hyaluronate; HA), VLA-4, VCAM-1, monocyte, rolling, leukocyte trafficking, parallel-plate flow chamber, cell adhesion

## Abstract

Leukocyte recruitment from blood into tissues involves sequential adhesive steps, including rolling and integrin-dependent arrest. VLA-4 can support firm adhesion and, in some settings, rolling interactions, whereas CD44–hyaluronan interactions have also been implicated in leukocyte rolling. Here, we used adhesion assays and parallel-plate flow chamber experiments to analyze CD44–hyaluronan-dependent monocyte interactions on ECV304 monolayers and to compare them with α4-integrin-sensitive adhesion on endothelial monolayers. WEHI 78/24 monocytoid cells interacted with ECV304 monolayers in a CD44- and hyaluronan-dependent manner, whereas adhesion to HMEC-1 and bEnd.3 monolayers was sensitive to α4-integrin blockade. Blocking CD44, adding soluble hyaluronan, or treating ECV304 monolayers with hyaluronidase reduced adhesion and rolling. Mixed primary human monocyte preparations also showed CD44-dependent adhesion and rolling on ECV304 monolayers. ECV304 cells are interpreted here not as endothelial cells, but as T24-derived, hyaluronidase-sensitive cellular monolayers useful for functional analysis of CD44–hyaluronan-dependent interactions. These findings support a substrate-dependent functional hierarchy in which CD44–hyaluronan-dependent monocyte rolling becomes detectable when α4-integrin-dependent adhesion is not dominant, while emphasizing the cell-model-based nature of the assay.

## 1. Introduction

Leukocyte recruitment to tissues proceeds through tethering, rolling, arrest, and transmigration under shear flow [1,2,3]. Rolling interactions are classically mediated by selectins, whereas firm adhesion and arrest largely depend on integrins such as α4β1 (VLA-4) and αLβ2 (LFA-1) [4,5]. In addition to classical selectin ligands and integrins, other glycosylated leukocyte surface molecules can contribute to adhesive interactions under flow. These include heat-stable antigen/CD24, which has been implicated in myeloid cell interactions with endothelial or platelet P-selectin under shear conditions [6,7], and CD44, a major hyaluronan receptor involved in leukocyte adhesion and trafficking [8,9,10,11]. Chemokine-mediated integrin activation can trigger rapid arrest under flow [12]. In monocytes, integrin-dependent adhesion pathways are particularly important for inflammatory recruitment to tissues [13,14,15]. After extravasation, monocytes differentiate into macrophages or monocyte-derived dendritic cells and contribute to tissue homeostasis and immune responses [16,17]. CD44–hyaluronan (HA; also termed hyaluronate) interactions can support rolling of lymphocytes under flow and usually generate weaker, more transient interactions than integrin-mediated adhesion [8,9]. CD44, originally described as the Hermes lymphocyte homing-associated antigen [10,11], has since been linked to leukocyte trafficking in several inflammatory models. Blocking CD44–hyaluronan interactions reduces leukocyte rolling and infiltration in experimental autoimmune uveoretinitis [18]. In lipopolysaccharide (LPS)-induced airway inflammation, CD44-deficient mice show impaired macrophage recruitment and reduced endothelial adhesion of cells, supporting a role for CD44 in monocyte/macrophage trafficking [19]. However, under physiological flow conditions, CD44-dependent rolling may be difficult to detect because it can be functionally masked by stronger integrin-mediated interactions, particularly those involving VLA-4/VCAM-1. Monocytes also differ from lymphocytes in their integrin repertoire and adhesive behavior under flow, raising the question of whether CD44–hyaluronan interactions can support monocyte rolling when α4-integrin-dependent adhesion is not dominant. To address this question, we compared monocyte interactions with cellular monolayers that differ in their functional adhesion properties. HMEC-1 and bEnd.3 monolayers were used as endothelial reference substrates with α4-integrin-sensitive adhesion, whereas T24-derived ECV304 monolayers were used as hyaluronidase-sensitive cellular substrates for the analysis of CD44–hyaluronan-dependent interactions. This design does not recreate the full complexity of vascular recruitment in vivo, but allows CD44-dependent rolling to be examined in a defined cell-based flow system.

Here, we show that CD44–hyaluronan-dependent rolling and transient adhesion can be detected on ECV304 monolayers when α4-integrin-dependent adhesion is not functionally dominant. The study therefore identifies a substrate-dependent functional hierarchy of receptor use in monocyte adhesion under flow.

## 2. Results

**Figure 1:**
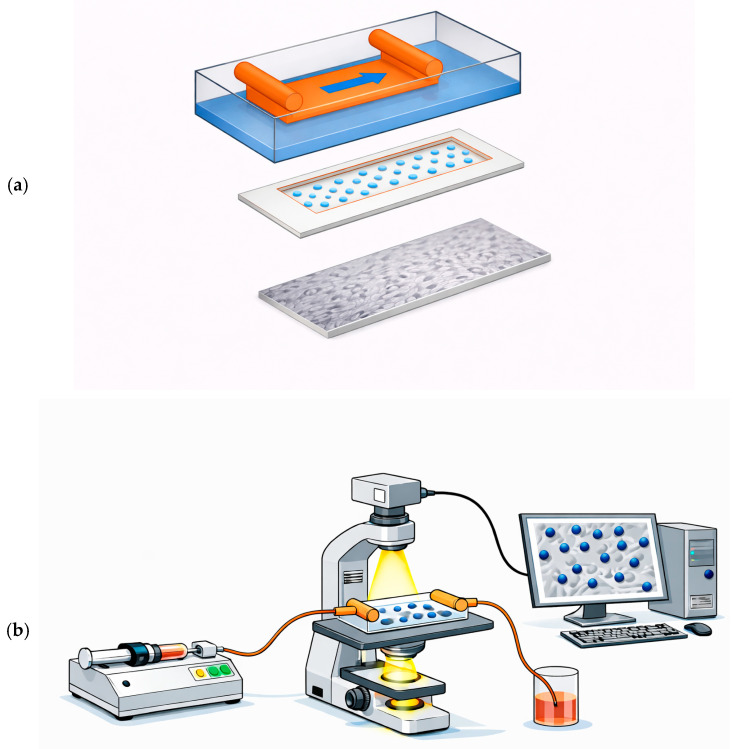
Parallel-plate flow chamber assay for analysis of CD44–hyaluronan-dependent monocyte interactions. (**a**) Schematic representation of the custom-made transparent parallel-plate flow chamber (gap height 250 µm; not drawn to scale). Confluent cellular monolayers are assembled in the chamber and exposed to defined laminar shear flow. The arrow indicates the direction of flow. (**b**) Monocytes are perfused through the chamber using a syringe pump and analyzed under controlled shear conditions. Rolling and transient adhesion are visualized on hyaluronan-presenting cellular monolayers (e.g., ECV304) under low shear stress using an inverted microscope equipped with video acquisition. This setup allows quantitative analysis of CD44–hyaluronan-mediated interactions under conditions of limited α4-integrin engagement. The figure was adapted from Eibl, with permission [20].

To determine whether monocytes can engage CD44–hyaluronan as a rolling mechanism when α4-integrin engagement is limited, we compared monocyte interactions with cellular monolayers that differ in adhesive properties. HMEC-1 and bEnd.3 monolayers were used as endothelial reference substrates in which adhesion was sensitive to α4-integrin blockade. ECV304 cells were used as a T24-derived, hyaluronidase-sensitive cellular monolayer to analyze CD44-dependent interactions. The flow chamber setup used for rolling measurements is summarized in Figure 1; the same parallel-plate flow chamber system, including the 250 µm chamber gap, was used to analyze chemokine-independent VLA-4/VCAM-1-mediated rolling and arrest of B16 melanoma cells under shear flow [20].

### 2.1. Distinct α4-Integrin- and CD44-Dependent Adhesion Pathways Are Revealed on Different Cellular Monolayers

**Figure 2:**
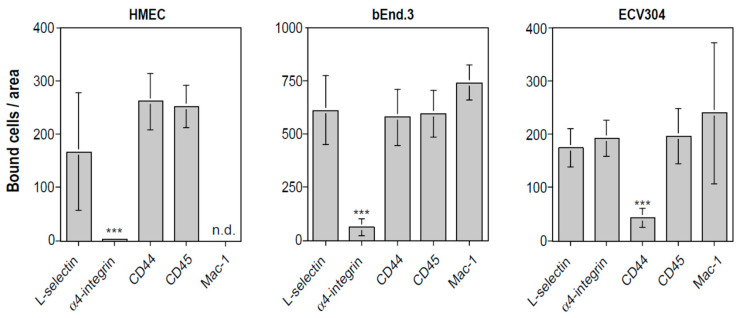
Distinct α4-integrin- and CD44-dependent adhesion pathways in monocytoid cells. Shaking adhesion assays were performed to quantify binding of WEHI 78/24 cells to HMEC-1, bEnd.3 and ECV304 monolayers in the presence of function-blocking antibodies. Adhesion to HMEC-1 and bEnd.3 was inhibited by blockade of α4 integrins (PS/2), whereas adhesion to ECV304 was selectively inhibited by anti-CD44. Control antibodies against L-selectin, CD45 and Mac-1 had no detectable effect. n.d., not determined. Data are shown as mean ± SD from *n* = 3 independent experiments. *** *p* < 0.001 versus CD45 control.

WEHI 78/24 monocytoid cells express major adhesion receptors including L-selectin and α4 integrins [21]. To assess whether these cells can engage CD44–hyaluronan independently of α4-integrin ligands, we compared their adhesion to HMEC-1, bEnd.3 and ECV304 monolayers in shaking adhesion assays (Figure 2). Adhesion to HMEC-1 and bEnd.3 monolayers was inhibited by function-blocking antibodies against α4 integrin, whereas the same treatment had no detectable effect on adhesion to ECV304 cells. Conversely, function-blocking anti-CD44 antibodies selectively inhibited adhesion to ECV304 monolayers, but not to HMEC-1 or bEnd.3. Control antibodies against L-selectin, CD45 and Mac-1 had no detectable effect.

These findings indicate that the same WEHI 78/24 cells can use different receptor pathways depending on the cellular substrate. On HMEC-1 and bEnd.3 monolayers, adhesion was sensitive to α4-integrin blockade, whereas CD44 blockade had no detectable inhibitory effect. In contrast, on ECV304 monolayers, α4-integrin blockade did not reduce adhesion, whereas CD44 blockade markedly inhibited adhesion. Thus, CD44-dependent adhesion was not absent from WEHI 78/24 cells, but became functionally detectable on a substrate in which α4-integrin-dependent adhesion was not dominant. This supports a substrate-dependent functional hierarchy of receptor use. ECV304 cells were originally described as endothelial cells but were later identified as derivatives of the T24 bladder carcinoma line. In the present study, they were used as T24-derived cellular monolayers for functional analysis of CD44–hyaluronan-dependent adhesion.

### 2.2. Rolling Interactions on ECV304 Monolayers Are Weaker than on bEnd.3 Monolayers

**Figure 3:**
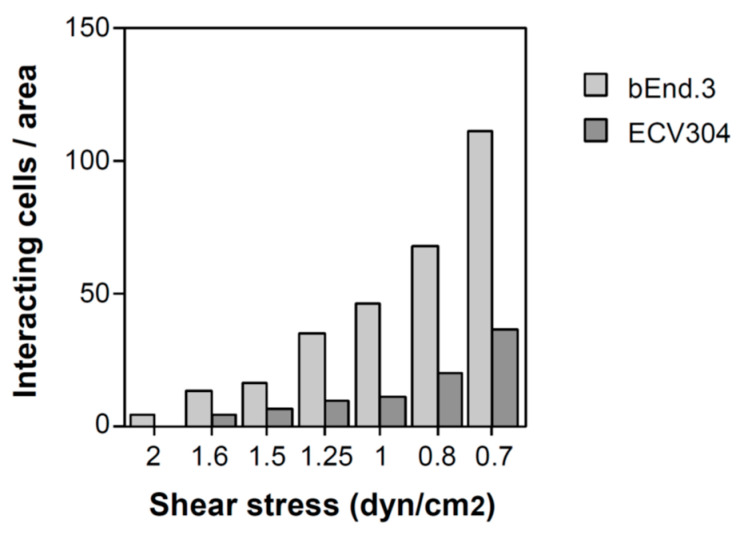
Rolling interactions of WEHI 78/24 cells under defined flow conditions. Rolling and adhesion of WEHI 78/24 cells were analyzed in a parallel-plate flow chamber on bEnd.3 and ECV304 monolayers at wall shear stresses between 0.7 and 2 dyn/cm^2^. Under identical flow conditions, bEnd.3 monolayers supported more interacting cells across the full range tested, whereas interactions on ECV304 monolayers were consistently lower. No statistical comparison is shown for this representative flow experiment. Data shown are representative of three independent experiments.

To compare rolling on bEnd.3 and ECV304 monolayers, WEHI 78/24 cells were analyzed in a parallel-plate flow chamber over a range of wall shear stresses from 0.7 to 2 dyn/cm^2^ (Figure 3). As shear stress decreased, interactions increased on both substrates. Across the range tested, bEnd.3 monolayers supported more interacting cells than ECV304 monolayers. Together with the antibody-blocking data in Figure 2, these findings are consistent with stronger α4-integrin-associated adhesion on endothelial monolayers and weaker CD44–hyaluronan-sensitive interactions on ECV304 monolayers. Because α4-integrin blockade was not included in the flow experiment shown in Figure 3, this experiment is interpreted as a comparison of substrate-dependent rolling behavior rather than as direct proof of receptor usage under flow.

### 2.3. CD44 Mediates Rolling and Arrest of WEHI 78/24 Cells on ECV304 Monolayers via Hyaluronan

**Figure 4:**
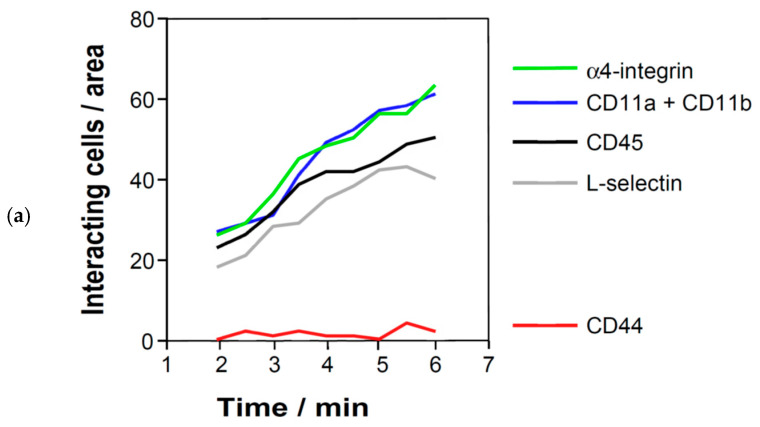

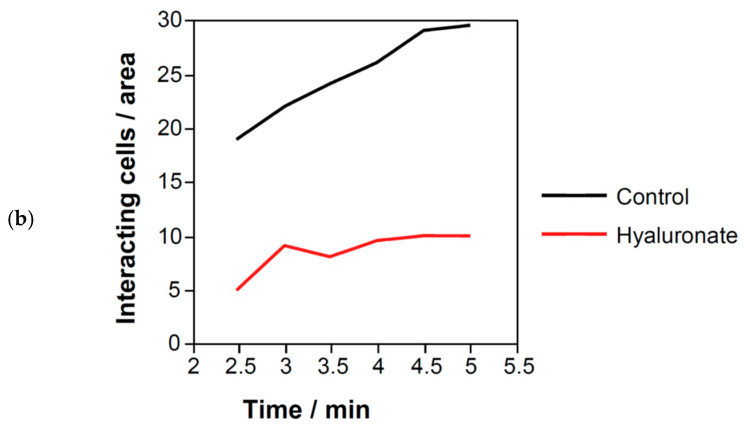
CD44-mediated rolling and arrest of WEHI 78/24 cells at low shear stress. (**a**) WEHI 78/24 cells were analyzed on ECV304 monolayers in parallel-plate flow chamber assays at 1 dyn/cm^2^ following pretreatment with function-blocking antibodies. Anti-CD44 markedly reduced rolling and firm arrest, whereas antibodies against L-selectin, α4 integrin, CD11a/CD11b, and CD45 had no detectable effect. (**b**) Pretreatment with soluble hyaluronan similarly inhibited rolling and arrest, supporting involvement of hyaluronan. Data shown are representative of three independent experiments.

To define the mechanism of rolling on ECV304 monolayers, WEHI 78/24 cells were analyzed under low shear stress of 1 dyn/cm^2^ (Figure 4). Under these conditions, cells progressively accumulated on the ECV304 surface over time through a combination of rolling and firm arrest. Pretreatment with a function-blocking anti-CD44 antibody markedly reduced both rolling and arrest, whereas antibodies against L-selectin, α4 integrin, CD11a/CD11b or CD45 had no detectable effect (Figure 4a). Pretreatment with soluble hyaluronan likewise reduced rolling and arrest, although inhibition was less complete than that observed with direct CD44 blockade (Figure 4b).

Together, these results show that CD44 blockade strongly reduces WEHI 78/24 rolling and arrest on ECV304 monolayers at low shear stress. The inhibition by soluble hyaluronan supports involvement of CD44–hyaluronan interactions in this assay.

### 2.4. WEHI 78/24 Cells Bind Soluble Hyaluronan via CD44

**Figure 5:**
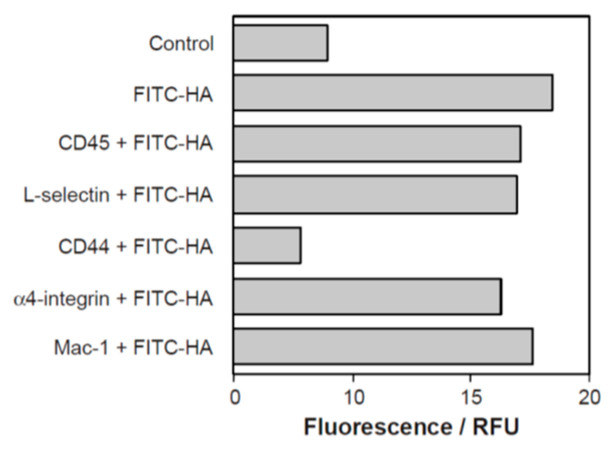
CD44 mediates soluble hyaluronan binding by WEHI 78/24 cells. Flow cytometric analysis demonstrated robust binding of FITC-labelled hyaluronan (FITC-HA) to WEHI 78/24 cells. Binding was abolished by a function-blocking anti-CD44 antibody, whereas control antibodies had no detectable effect. Representative histograms from three independent experiments are shown.

To determine whether WEHI 78/24 cells directly bind hyaluronan via CD44, FITC-labelled hyaluronan was analyzed by flow cytometry (Figure 5). Untreated WEHI 78/24 cells showed strong hyaluronan binding, whereas pretreatment with a function-blocking anti-CD44 antibody abolished binding and reduced fluorescence to baseline levels. Control antibodies had no detectable effect. These data confirm that CD44 on WEHI 78/24 cells functions as the principal hyaluronan receptor in this system and support the interpretation that rolling on ECV304 monolayers is mediated through CD44–hyaluronan interactions.

### 2.5. Surface-Associated Hyaluronan on ECV304 Monolayers Supports Adhesion of WEHI 78/24 Cells

**Figure 6:**
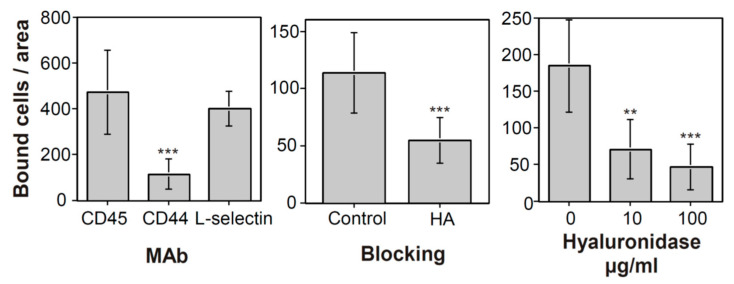
CD44–hyaluronan-dependent adhesion of WEHI 78/24 cells to ECV304 monolayers. Adhesion of WEHI 78/24 cells to ECV304 monolayers was assessed in static assays following pretreatment with function-blocking anti-CD44 antibodies (**left**), soluble hyaluronan (**middle**), or hyaluronidase treatment of the monolayer (**right**). Anti-CD44 and soluble hyaluronan inhibited adhesion, and hyaluronidase reduced adhesion in a dose-dependent manner. Control antibodies, including anti-L-selectin, had no detectable inhibitory effect. Data are shown as mean ± SD from *n* = 3 independent experiments. ** *p* < 0.01, *** *p* < 0.001 versus the corresponding control in each panel.

To test whether hyaluronan presented by ECV304 cells contributes to monocyte adhesion, static adhesion assays were performed in the presence of blocking antibodies, soluble hyaluronan, or hyaluronidase treatment (Figure 6). Binding of WEHI 78/24 cells to ECV304 monolayers was inhibited by anti-CD44, but not by antibodies against CD45 or L-selectin (Figure 6, left). Soluble hyaluronan partially reduced binding (Figure 6, middle), consistent with competition for CD44. Enzymatic treatment of ECV304 monolayers with increasing concentrations of hyaluronidase led to a dose-dependent reduction in adhesion (Figure 6, right).

These results show that adhesion of WEHI 78/24 cells to ECV304 monolayers is sensitive to CD44 blockade and to perturbation of hyaluronan. Although surface hyaluronan on ECV304 cells was not directly quantified in these experiments, the inhibition by soluble hyaluronan and by hyaluronidase treatment supports a functional role of hyaluronan in the observed CD44-dependent adhesion.

### 2.6. Primary Human Monocytes Use CD44 to Bind Hyaluronan-Presenting ECV304 Monolayers

**Figure 7:**
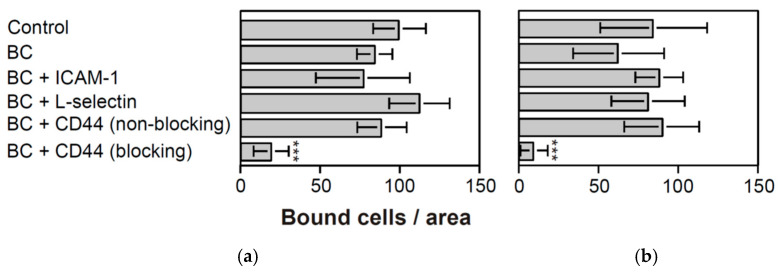
CD44-dependent adhesion of primary human monocytes to ECV304 monolayers. Human monocyte adhesion to ECV304 monolayers was assessed under (**a**) static and (**b**) shaking conditions. Control samples were analyzed without blocking antibodies. BC denotes the basal blocking cocktail consisting of antibodies against P-selectin, β1 integrins, and β2 integrins. Additional antibodies were included as indicated: ICAM-1, L-selectin, CD44 (non-blocking), or CD44 (blocking). Only the function-blocking anti-CD44 antibody markedly reduced adhesion, whereas the other added antibodies did not produce comparable inhibition. Data are shown as mean ± SD from *n* = 3 independent experiments. *** *p* < 0.001 versus the BC + CD44 (non-blocking) antibody control.

To determine whether the CD44-dependent interaction observed in WEHI 78/24 cells is conserved in primary cells, human peripheral blood monocytes were analyzed on ECV304 monolayers under static and shaking conditions (Figure 7). Only function-blocking anti-human CD44 antibodies inhibited monocyte adhesion, whereas non-blocking anti-CD44 antibodies such as HERMES-3 and additional control antibodies had no detectable effect. A basal blocking cocktail directed against P-selectin, β1 integrins and β2 integrins did not reduce adhesion compared with untreated controls, and addition of anti-ICAM-1, anti-L-selectin, or non-blocking anti-CD44 likewise did not produce comparable inhibition.

These findings indicate that mixed primary human monocyte preparations can also engage CD44-dependent adhesion on ECV304 monolayers. The data support conservation of the CD44-sensitive interaction in primary human cells, while not distinguishing between monocyte subsets.

### 2.7. Primary Human Monocytes Also Roll on ECV304 Monolayers via CD44 Under Low Shear

**Figure 8:**
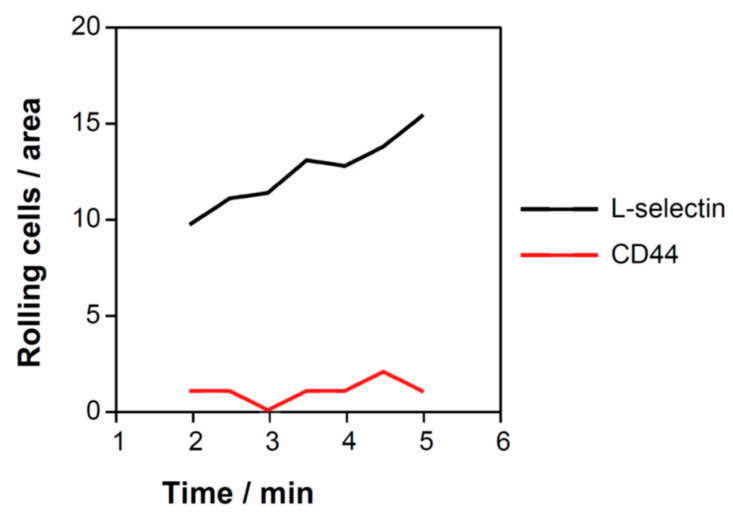
CD44 mediates rolling of primary human monocytes under low shear. Primary human monocytes were analyzed on ECV304 monolayers in parallel-plate flow chamber assays at 1 dyn/cm^2^. A function-blocking anti-CD44 antibody markedly reduced rolling interactions, whereas control antibodies had no detectable effect. These findings show that CD44-dependent rolling on ECV304 monolayers was also detectable in human monocyte preparations.

To examine whether the CD44-dependent interaction was also detectable in primary cells, human peripheral blood monocytes were analyzed under flow (Figure 8). At low shear stress of 1 dyn/cm^2^, human monocytes displayed robust rolling interactions on ECV304 monolayers. Pretreatment with a function-blocking anti-CD44 antibody markedly reduced rolling, whereas control antibodies had no detectable effect.

Thus, CD44-dependent rolling on ECV304 monolayers was also detected in primary human monocyte preparations. These experiments extend the WEHI 78/24 findings to primary human cells, but they should be interpreted as results obtained with mixed monocyte preparations rather than with defined CD14/CD16 monocyte subsets.

## 3. Discussion

CD44–hyaluronan interactions have previously been implicated in leukocyte adhesion and rolling under inflammatory and tissue-specific conditions [9,21,22,23]. The present study asked whether CD44–hyaluronan-dependent interactions can support monocyte rolling under shear flow on a cellular monolayer in which α4-integrin-dependent adhesion is not functionally dominant. The data support this conclusion for ECV304 monolayers: CD44 blockade, soluble hyaluronan, and hyaluronidase treatment reduced adhesion and rolling, whereas α4-integrin blockade did not measurably reduce interactions with this substrate.

The comparison of WEHI 78/24 adhesion across different monolayers supports a substrate-dependent functional hierarchy of receptor use. On HMEC-1 and bEnd.3 monolayers, α4-integrin blockade strongly reduced adhesion, whereas CD44 blockade had no detectable inhibitory effect. Conversely, on ECV304 monolayers, α4-integrin blockade did not reduce adhesion, whereas CD44 blockade markedly inhibited adhesion. Thus, CD44-dependent adhesion was not absent from WEHI 78/24 cells, but was functionally masked by dominant α4-integrin-dependent adhesion and became detectable when α4-integrin engagement was limited. This should be interpreted as a functional hierarchy revealed by different cellular substrates, rather than as a complete molecular dissection of all receptor–ligand pairs on a single engineered surface. ECV304 cells were originally introduced as endothelial-like cells but are now recognized as derivatives of the T24 bladder carcinoma line. They should therefore not be interpreted as authentic vascular endothelial cells. In this study, ECV304 cells served as a T24-derived, hyaluronidase-sensitive cellular monolayer for functional analysis of CD44–hyaluronan-dependent monocyte interactions. This use is informative for a cell-based adhesion assay, but it limits extrapolation to intact vascular endothelium in vivo.

The weaker rolling observed on ECV304 monolayers compared with bEnd.3 monolayers is consistent with the transient nature of CD44-mediated adhesion under flow [14,24]. A possible explanation is receptor topography. As von Andrian and colleagues showed, efficient tethering and rolling under flow depend on the spatial presentation of adhesion receptors on leukocyte microvilli [25]. CD44 was largely excluded from microvillous tips in the leukocyte subsets analyzed in that study, whereas selectins were enriched at microvillous tips. Although monocytes were not specifically analyzed there, this provides a plausible structural explanation for why CD44–hyaluronan interactions are more readily detected under low-shear conditions and are weaker than classical selectin- or integrin-based interactions.

The present findings are consistent with previous in vivo observations linking CD44 to leukocyte recruitment. CD44–hyaluronan interactions have been implicated in leukocyte rolling and tissue recruitment in uveoretinitis, inflamed liver sinusoids, and vascular inflammatory models [18,21,22]. In inflammatory airway disease, CD44-deficient mice showed impaired macrophage recruitment and reduced endothelial adhesion of macrophages [19]. The present flow-chamber data complement these observations by showing that CD44-sensitive rolling can be detected in a defined cell-based assay, but they do not by themselves establish the relative importance of CD44 compared with VLA-4 in vivo.

CD44 may also interact with non-hyaluronan ligands, including selectins, and cholesterol-dependent redistribution of CD44 has been shown to modulate monocyte rolling in the context of E-selectin [26]. In the present system, however, rolling was reduced by soluble hyaluronan and by hyaluronidase treatment of the monolayer, supporting involvement of hyaluronan. Activated ECV304 cells have also been reported to express VCAM-1 under certain conditions [27]. In our assays, α4-integrin blockade did not reduce adhesion or rolling on ECV304 monolayers, whereas CD44 blockade, soluble hyaluronan, and hyaluronidase treatment did. Thus, the observed interactions were functionally CD44–hyaluronan-sensitive rather than α4-integrin-sensitive.

The human monocyte experiments support the relevance of the mechanism in primary cells, but they also have limitations. Because primary human monocytes were analyzed as mixed preparations, the present study does not assign CD44-dependent rolling to classical, intermediate, or non-classical CD14/CD16-defined monocyte subsets. Future experiments should also address whether CD44–hyaluronan binding by primary human monocytes is dynamically regulated by chemokines. In our unpublished pilot observations, short exposure to CXCL12/SDF-1 increased detectable hyaluronan binding by primary human monocytes, including after exposure for as little as 1 min before fixation for flow cytometric analysis, whereas donor-matched lymphocytes did not show comparable induction. These observations were not part of the present study and should be confirmed in larger donor cohorts, including defined CD14/CD16 monocyte subsets and flow-based functional assays. Several additional limitations should be noted. The study relies on historical flow-chamber experiments and functional perturbation with blocking antibodies, soluble hyaluronan, and hyaluronidase rather than on engineered gain- or loss-of-function systems on a single defined substrate. The experiments did not directly quantify VCAM-1, ICAM-1, P-selectin, or surface hyaluronan on the monolayers in parallel with the flow assays, and they did not provide a full immunophenotypic comparison of WEHI 78/24 cells and human monocyte subsets. In addition, primary human monocyte yield varied between donors, and recording duration in some flow experiments was limited by the available cell number. For this reason, some flow data are presented as representative independent experiments showing reproducible qualitative behavior rather than as pooled time-course statistics. The data should therefore be interpreted as functional evidence for CD44–hyaluronan-dependent monocyte rolling on ECV304 monolayers, not as a complete molecular dissection of all receptor–ligand combinations involved in monocyte recruitment.

Within these limits, the model may still be useful for analyzing monocyte interactions with structurally altered or tumor-associated cellular surfaces under flow. This is consistent with recent flow-chamber work showing that tumor cells can engage defined adhesion receptor–ligand pathways, such as VLA-4/VCAM-1, during rolling and arrest under shear conditions [20]. Tumor-associated vasculature and abnormal vessel-like structures, including vasculogenic mimicry, can create non-canonical cellular interfaces and altered shear conditions [22,23,24,25,28,29]. Although the present study does not model tumor vasculature directly, the use of a T24-derived ECV304 monolayer provides a simplified cellular surface on which CD44–hyaluronan-sensitive monocyte rolling can be examined. Altered hyaluronan synthesis and accumulation are common features of many tumors [26], and CD44-related adhesion mechanisms have also been linked to tumor cell dissemination and metastasis-associated phenotypes [27,30,31,32]. These connections should be considered hypothesis-generating rather than direct conclusions from the present experiments.

## 4. Materials and Methods

### 4.1. Cells and Reagents

bEnd.3 cells (American Type Culture Collection [ATCC] CRL-2299), a mouse brain endothelial cell line derived from primary brain endothelial cells transformed with polyomavirus middle T antigen [33], were maintained in complete DMEM (cDMEM; DMEM supplemented with 5% fetal bovine serum [endotoxin < 10 pg/mL; Gemini Scientific, West Sacramento, CA, USA] and 5% Fetal Clone [Hyclone Laboratories, Logan, UT, USA]). Cells were used between passages 22 and 30.

ECV304 cells (ATCC CRL-1998) were originally described as spontaneously transformed human endothelial cells [34], but were later shown to be derived from the T24 bladder carcinoma line [35,36,37,38]. Cells were used as historically maintained laboratory stocks corresponding to the originally distributed ECV304 line. In the present study, ECV304 cells were therefore not used as a surrogate for authentic vascular endothelium, but as a T24-derived cellular monolayer providing a hyaluronidase-sensitive, hyaluronan-presenting surface for functional analysis of CD44-dependent interactions. ECV304 cells were maintained in M199 medium supplemented with 10% fetal bovine serum [endotoxin < 10 pg/mL; Gemini Scientific].

HMEC-1 human dermal microvascular endothelial cells (ATCC CRL-3243) [39] were maintained in MCDB-131 medium supplemented with 10 ng/mL epidermal growth factor, 1 µg/mL hydrocortisone and 10% fetal bovine serum [endotoxin < 10 pg/mL; Gemini Scientific].

WEHI 78/24 murine monocytoid cells [40] were obtained as a gift from R. Coffman (DNAX Research Institute, Palo Alto, CA, USA), cultured in cDMEM and sub-cultured 36 h before use. WEHI 78/24 cells were used as a murine monocytoid model cell line. Previous work described WEHI 78/24 as a monocytoid/accessory-cell-like line [40]. In the present study, receptor involvement was assessed functionally by antibody blockade and by FITC–hyaluronan binding rather than by comprehensive immunophenotyping. Functional CD44 expression was confirmed by FITC–hyaluronan binding and CD44 blockade, whereas α4-integrin involvement was inferred from PS/2-sensitive adhesion to HMEC-1 and bEnd.3 monolayers. Direct comparison with human CD14/CD16 monocyte subsets was not performed.

Primary human monocytes were isolated from peripheral blood samples from healthy adult donors using standard previously published procedures. Quality controls included collection of samples from each major step, followed by FACS analysis to monitor enrichment and sample quality. All samples were obtained with written informed consent; no identifying information was associated with the samples. Human monocytes were used as mixed primary monocyte preparations and were not further separated into CD14/CD16-defined subsets. FITC-labelled hyaluronan (FITC-HA) was prepared fresh (Sigma; St. Louis, MO, USA; cat. no. H5388), tested (Figure S1) and stored in aliquots as described previously [41].

Antibodies used in this study were as follows: HERMES-3 (mouse IgG2a, anti-human CD44) [42]; 84H10 (anti-human ICAM-1); L133 (anti-human CD31); TY1138 (anti-human VCAM-1); WAPS1.2 (anti-human P-selectin) [43]; DREG56 (mouse IgG1, anti-human L-selectin) [43]; 9B5 (anti-human CD44); IM7.8.1 or TJB1.7 (anti-mouse CD44, depending on experiment); Mel-14 (anti-mouse L-selectin) [44]; PS/2 (rat IgG2b, anti-mouse α4 integrin) [45]; MI/70 (anti-mouse αM/Mac-1) [46]; TIB213 (anti-mouse αL); and 30G12 (rat IgG2a, anti-mouse CD45) [47]. Additional antibodies used in specific experiments are indicated in the corresponding figure legends.

### 4.2. Monolayer Activation

HMEC-1, bEnd.3 or ECV304 monolayers were grown to confluence and treated with recombinant human TNF-α (1 ng/mL; R&D Systems, Minneapolis, MN, USA) for 18 h before use in adhesion assays.

### 4.3. Hyaluronidase Treatment

Monolayers were washed extensively in DMEM and incubated with hyaluronidase (10 or 100 µg/mL) in DMEM containing 10 mM HEPES for 1 h at 37 °C. Monolayers were then washed twice to remove enzyme and used immediately in binding or flow assays to prevent synthesis of new hyaluronate.

### 4.4. Flow Cytometry

Binding of soluble FITC-labelled hyaluronan to WEHI 78/24 cells was analyzed by flow cytometry. Cells were incubated with FITC-hyaluronan in the presence or absence of blocking antibodies under standard conditions and analyzed on a FACScan (Becton, Dickinson and Company, Franklin Lakes, NJ, USA) using CellQuest software version 3.1. Experiments were repeated at least three times with comparable results.

### 4.5. Static and Shaking Adhesion Assays

Monolayer-forming cell lines were seeded into 1 cm^2^ wells of 8-well Lab-Tek chamber slides (Nunc Inc., Naperville, IL, USA) and grown to confluence for 2–3 days. Monolayers were treated with TNF-α (1 ng/mL) for 18 h, washed twice with assay buffer and left in 100 µL/well before addition of WEHI 78/24 cells or primary human monocytes. Before the assay, WEHI 78/24 cells were resuspended at 2 × 10^6^ cells/mL and preincubated with saturating concentrations of antibody (10 µg/mL) for 15 min at room temperature or with soluble hyaluronan (400 µg/mL) for 30 min at room temperature. A total of 2 × 10^5^ cells was added in 100 µL/well for a final volume of 200 µL. Assays were performed at room temperature with continuous rocking. Chambers were rotated by 90° after 10 min to facilitate even binding. After 20 min, the chamber top and gasket were removed, slides were dipped twice in PBS to remove non-adherent cells and fixed in 1.5% glutaraldehyde in PBS. Bound cells were quantified by manual counting under light microscopy. The mean number of bound cells per field or well was determined as indicated in the corresponding figures. Primary human monocyte adhesion assays were performed analogously under static or shaking conditions as specified in the figure legends.

### 4.6. Monocyte–Hyaluronate Binding Assay

Hyaluronan (3.3 mg/mL in PBS, 0.1% BSA) was diluted in PBS to 400 µg/mL. A volume of 200 µL was added to 1 cm^2^ wells of 8-well Lab-Tek chamber slides (Nunc Inc., Naperville, IL, USA) and allowed to bind overnight at 4 °C. Remaining binding sites were blocked for 30 min at room temperature with PBS containing 1% BSA. Slides were washed twice with assay buffer and left in 100 µL/well before addition of cells. During the blocking step, WEHI 78/24 cells were preincubated with antibodies (10 µg/mL) for 15 min at room temperature. After incubation, slides were examined by light microscopy and the mean number of bound cells was determined.

### 4.7. Laminar Flow Assays

Parallel-plate laminar flow assays were performed using a custom-made flow chamber (Figure 1a) based on established flow-chamber designs [48,49,50]. Monolayer cells were grown to confluence on glass slides (Superfrost Microscope Slides, Erie Scientific, Portsmouth, NH, USA) and assembled in a parallel-plate chamber with a 250 µm gap thickness, generating uniform wall shear stress across the monolayer. The chamber was mounted on the stage of an inverted phase-contrast microscope. WEHI 78/24 cells or primary human monocytes were resuspended at 2 × 10^6^ cells/mL in assay buffer and perfused through the chamber with a Harvard syringe pump at defined flow rates (Figure 1b). Wall shear stress was calculated from chamber geometry and volumetric flow rate, assuming a viscosity of 1.0 cP. Experiments were performed over a shear-stress range of 0.7–2 dyn/cm^2^, as indicated in the results and figure legends. For low-shear rolling assays, a wall shear stress of 1 dyn/cm^2^ was used. The flow rate was stepped down, where indicated, to allow measurement of rolling and firm adhesion at different shear stresses. Two minutes were allowed for equilibration after each change in flow. Interacting cells were counted every 30 s for approximately 2–4 min. Cell behavior, including rolling and arrest, was recorded by video microscopy and quantified by analysis of recorded images. For experiments with primary human monocytes, the available cell numbers varied between donors because blood sample volume and monocyte yield differed. In some flow experiments, especially at higher flow rates, the available cell suspension was depleted within a few minutes. Therefore, recording duration and the number of evaluable time intervals were not identical in all independent experiments. Flow experiments with comparable qualitative outcomes but unequal recording duration were therefore presented as representative independent experiments rather than pooled time-course analyses.

### 4.8. Statistics

Data are presented as mean ± SD from at least three independent experiments unless otherwise indicated. When multiple microscopic fields or wells were analyzed within one experiment, these technical replicates were averaged before statistical analysis so that each independent experiment contributed one value. Comparisons involving more than two experimental groups were analyzed using one-way ANOVA followed by Dunnett’s multiple-comparisons test when several treatments were compared with a single control. Two-group comparisons, including soluble hyaluronan competition assays and selected flow experiments with one treatment condition, were analyzed using an unpaired two-tailed Student’s *t*-test. A *p* value < 0.05 was considered statistically significant. Because several datasets derive from historical flow-chamber experiments with limited sample size and variable recording duration, statistical results are interpreted together with effect size, reproducibility across independent experiments, and biological consistency. Flow experiments shown as representative recordings were not used for pooled inferential statistics unless identical quantitative sampling was available across independent experiments.

### 4.9. Study Approval and Human Samples

The study was conducted in accordance with institutional guidelines and ethical standards applicable at the time the human samples were obtained. Written informed consent was obtained from all healthy adult donors. No identifying information was associated with the samples.

### 4.10. AI Tool Disclosure

AI-assisted tools (ChatGPT, using GPT-3.5 and GPT-5.5 Thinking models; OpenAI, San Francisco, CA, USA) were used for language editing and 3D-refinement of two author-generated schematic illustrations (Figure 1a,b). No AI tools were used for data generation, analysis, or interpretation. All scientific content and conclusions were generated and verified by the authors.

## 5. Conclusions

This study shows that CD44–hyaluronan interactions can support monocyte rolling and transient adhesion on ECV304 monolayers under low-shear flow. Adhesion to HMEC-1 and bEnd.3 monolayers was sensitive to α4-integrin blockade, whereas interactions with ECV304 monolayers were reduced by CD44 blockade, soluble hyaluronan, and hyaluronidase treatment. Similar CD44-sensitive rolling was observed in mixed primary human monocyte preparations. Together, these findings support a substrate-dependent functional hierarchy in which CD44–hyaluronan-dependent rolling is functionally masked when α4-integrin-dependent adhesion is dominant, but becomes detectable when α4-integrin engagement is limited. The model may therefore be useful for studying monocyte interactions with structurally altered or tumor-associated cellular surfaces under flow.

## Data Availability

The original contributions presented in this study are included in the article/Supplementary Materials. Further inquiries can be directed to the corresponding author.

## Supplementary Materials

The following supporting information can be downloaded at: https://www.mdpi.com/article/10.3390/ijms27125358/s1.
