# Supplementary material for: CD44–Hyaluronan-Dependent Monocyte Rolling
Source: Int J Mol Sci. 2026 Jun 13;27(12):5358. doi: 10.3390/ijms27125358 (PMC13299323; doi:10.3390/ijms27125358)

Figure S1. Positive control: FITC–hyaluronan (FITC-HA) binds to BW5147 cells (upper panel). In contrast, spleen leukocytes show minimal or no binding of FITC–hyaluronan (lower panel).

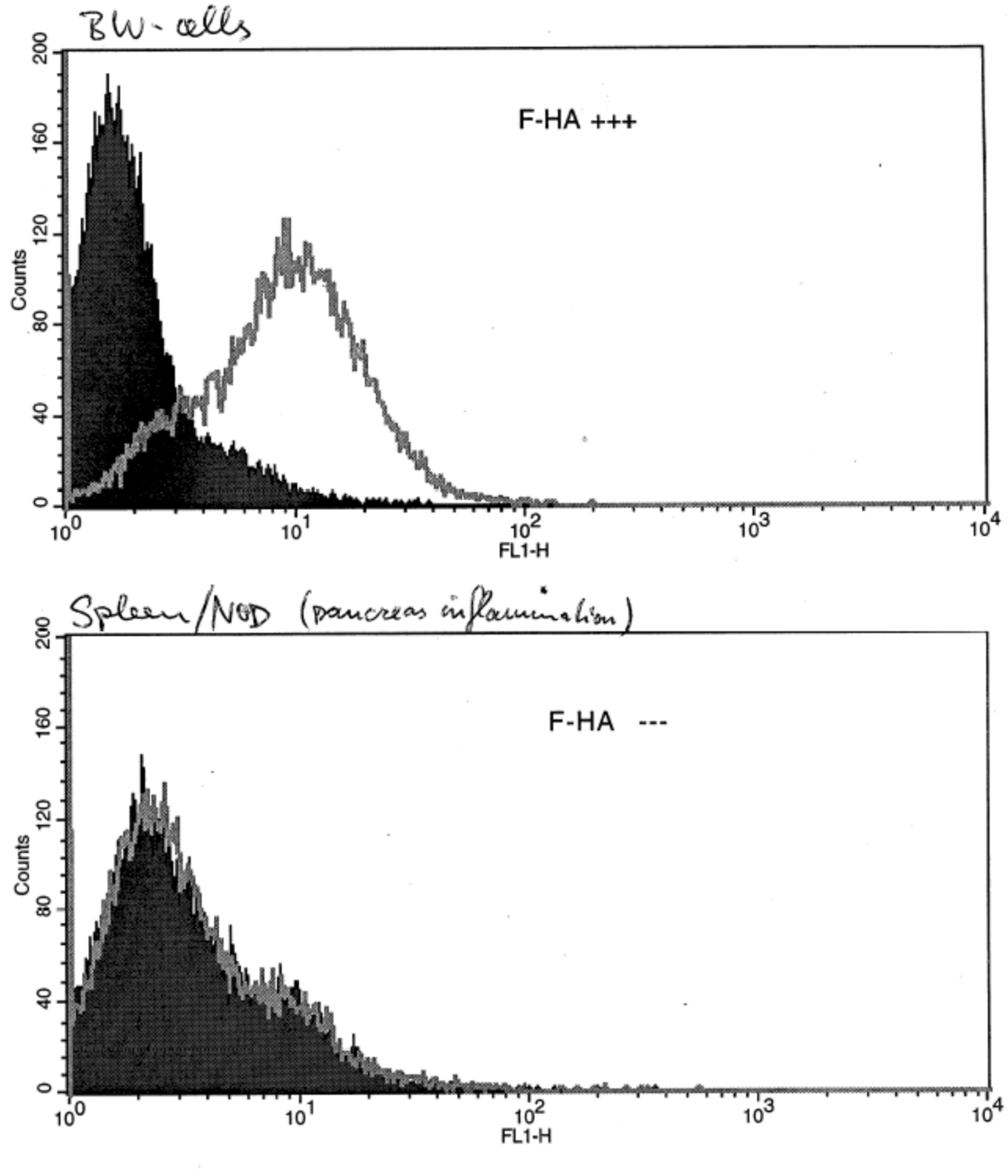

Supplement: Supplementary file 1 [file ijms-27-05358-s001.zip › ijms-4351092-supplementary.pdf]
